# Editorial: Functional Relevance of Tetraspanins in the Immune System

**DOI:** 10.3389/fimmu.2019.01714

**Published:** 2019-07-24

**Authors:** Carlos Cabañas, María Yáñez-Mó, Annemiek B. van Spriel

**Affiliations:** ^1^Department of Cell Biology and Immunology, Centro de Biología Molecular Severo Ochoa (CSIC-UAM), Madrid, Spain; ^2^Department of Immunology, Ophthalmology and Otorhinolaryngology (IO2), Faculty of Medicine, Universidad Complutense, Madrid, Spain; ^3^Instituto de Investigación Sanitaria Hospital 12 de Octubre (i+12), Madrid, Spain; ^4^Departamento de Biología Molecular, CBM-SO, UAM/IIS-IP, Madrid, Spain; ^5^Department of Tumor Immunology, Radboud Institute for Molecular Life Sciences, Radboud University Medical Center, Nijmegen, Netherlands

**Keywords:** tetraspanin, immune cell, infection, vaccine, cell membrane

Tetraspanins, members of the superfamily of four-transmembrane proteins, are evolutionary highly conserved membrane proteins that function as membrane-organizers (1–3). Immune cells express thousands of different membrane proteins (including adhesion receptors, uptake receptors, major histocompatibility molecules, enzymes, cytokine receptors, and others) that all need to be correctly localized in time and space at the cell surface. Tetraspanins specifically interact *in cis* with various immune receptors by forming multimolecular complexes (tetraspanin-enriched microdomains, TEMs) that can initiate immune cell signaling (4, 5). Thereby, tetraspanins control fundamental immune cell functions, including adhesion, pathogen uptake, immunological synapse formation, and proliferation. Tetraspanin-deficiency in mouse models and patients results in different immunological defects (6). This Review Topic provides a timely overview of the biological importance of tetraspanin-induced membrane organization in the immune system and the latest insights in targeting tetraspanins as novel drugs for infectious disease and cancer.

Immune cells are the fastest migrating cells in our body that depend on tight cooperation of different adhesion molecules (integrins, selectins, immunoglobulin superfamily members) and chemokine receptors to patrol for pathogens and reach inflammatory sites. For integrins, it has been well-established that clustering (avidity) and conformational change (affinity) both underlie activation. Tetraspanins are well-defined interaction partners for integrins (2, 7) that control adhesion and migration of lymphocytes, dendritic cells (8), and neutrophils (9). Yeung et al. discuss the functional roles of tetraspanins in leukocytes and endothelial cells during transmigration from the circulation into tissues. Most tetraspanins (CD9, CD37, CD81, and CD151) promote lymphoid and myeloid cell adhesion and migration through functional interaction with β1 and β2 integrins. In this regard, CD9 regulates the adhesive capacity of integrin α5β1 by modulating its association with the membrane protease ADAM17 on the cell surface (Machado-Pineda et al.). CD9 is also required for myeloid cell migration in a murine colitis model shown by decreased neutrophil and macrophage infiltration in colonic tissue from CD9-deficient mice (Saiz et al.). In contrast, tetraspanin CD82 in dendritic cells reduces cell motility through regulation of cytoskeletal proteins (e.g., RhoA). It is not known whether CD82 directly interacts with Rho GTPases, in line with the identified interaction between CD81 and Rac1 (10), or alternatively that CD82 regulates the cytoskeleton via interacting with ezrin/radixin/moesin (ERM) proteins. Besides directly interacting with adhesion molecules, tetraspanins have been reported to control the activity of membrane metalloproteases that can induce cleavage of adhesion receptors. For example, CD9 inhibits the shedding activity of ADAM17 and thereby supports ALCAM-dependent adhesion in antigen-presenting cells as discussed by Reyes et al. In line with this, tetraspanins of the TspanC8 subgroup, containing eight cysteine residues in their large extracellular loops, are required for ADAM10 exit from the endoplasmic reticulum and trafficking to the cell surface or other membrane compartments (11, 12). ADAM10 is well characterized as the ligand-dependent activator of Notch proteins, and Mike Tomlinson and colleagues discuss how TspanC8 members (Tspan5, Tspan10, Tspan14, Tspan15, Tspan17, and Tspan33) may control ADAM10 activity on myeloid and lymphoid cells in a specific manner (Matthews et al.). The most highly expressed TspanC8 in human and mouse T cells is Tspan14, followed by Tspan5 and Tspan17. Since both Tspan14 and Tspan5 promote Notch signaling, it is hypothesized that ADAM10 will have a major role in thymocyte development. Human and mouse B cells express high levels of Tspan33 and Tspan14, respectively, which may regulate Notch2 signaling and/or shedding of other ADAM10 ligands, such as CD23 (the IgEεRII). Some of these questions can be addressed by investigating newly generated Tspan14 and Tspan5-knockout mice. The authors also propose that targeting individual TspanC8 members may provide a novel therapeutic approach for ADAM10-associated diseases (leukemia, asthma, atherosclerosis, and Alzheimer's disease) without the toxicity of global ADAM10 inhibition.

Tetraspanins on antigen-presenting cells control multiple different functions, including cell migration, pathogen uptake, MHC trafficking, immunological synapse formation and antigen-presentation as reviewed by the group of Saiz et al. In B cells, tetraspanins (CD37, CD53, CD81) are essential for B cell receptor signaling, antibody production and cytokine secretion (Zou et al.). Interestingly, CD37-deficiency leads to spontaneous B cell lymphoma formation in mice, and patients with CD37-deficient B cell lymphomas have inferior clinical outcome than patients with CD37-positive lymphomas (13).

The pathogenesis of different infectious diseases is also influenced by different tetraspanin proteins (14). Besides adhesion and signaling, tetraspanins have been related to different membrane fusion events. The group of Peter Monk has explored the role of different tetraspanin members in membrane fusion of monocytic cells in response to *Mycobacterium tuberculosis* infection (Champion et al.). Tetraspanins can also interfere with different stages of the virus replication cycle. Florin and Lang evaluate how viruses exploit TEMs for viral entrance into cells, and subsequent budding and egress. Some viruses use specific tetraspanins as receptors (for example CD151-HPV, CD81-HCV) and by compartmentalizing host entry factors. In addition, viral envelope proteins accumulate in TEMs during morphogenesis, and induce large assemblies of tetraspanins and viral transmembrane proteins to facilitate budding. For example, tetraspanins (CD9, CD63, CD81, CD82) can be incorporated into the enveloping membrane of virions, such as HIV, feline immunodeficiency virus, influenza or hepatitis A virus, indicating that TEMs directly stimulate virus budding and exit. New evidence shows that HIV-1 replication is stimulated by CD81 through its direct interaction with the deoxynucleoside triphosphate phosphohydrolase SAMHD1 (15). Suárez et al. elaborate on the mechanisms underlying tetraspanin regulation of HIV-1 replication, which may be exploited to develop tetraspanin-based therapeutics as a novel strategy to restrict HIV-1 infection.

Given the plethora of immune functions that are controlled by tetraspanins, it is maybe not surprising that tetraspanins are important in anti-tumor immune responses. Even though this field is rather unexplored, tetraspanins expressed by immune suppressive cells (such as regulatory T cells, myeloid-derived suppressor cells) can control immune responses within the tumor microenvironment as discussed by Schaper and van Spriel. In addition, tetraspanins have been shown to modulate cancer metastasis indirectly through exosomes, and by regulating cellular interactions in the immune system as reflected on by Vences-Catalán and Levy.

Taken together, although broad in function, the underlying mechanism by which different tetraspanins accomplish their function is highly similar. Through their lateral molecular interactions with immune receptors, enzymes and/or signaling proteins, tetraspanins are in charge of organizing the protein landscape at the plasma membrane of immune cells. Evidence is now accumulating that these protein interactions are dynamic and likely change upon cell activation. Future research should provide better insight into the (1) specificity versus redundancy of individual tetraspanins on immune cell function, and (2) molecular mechanisms underlying TEM formation and coupling to signaling transduction pathways. The potency of targeting tetraspanins is currently under investigations at the (pre-)clinical level as novel therapeutics for cancer, infectious diseases and auto-immunity disorders (16, 17).

## Author Contributions

CC, MY-M, and AS have contributed to the writing of this editorial article. They are the three authors for correspondence of this editorial.

### Conflict of Interest Statement

The authors declare that the research was conducted in the absence of any commercial or financial relationships that could be construed as a potential conflict of interest.
